# Cognitive Function and Consumption of Fruit and Vegetable Polyphenols in a Young Population: Is There a Relationship?

**DOI:** 10.3390/foods8100507

**Published:** 2019-10-17

**Authors:** Juan Ángel Carrillo, M Pilar Zafrilla, Javier Marhuenda

**Affiliations:** Faculty of Health Sciences, Universidad Católica de San Antonio, 30107 Murcia, Spain; jacarrillo4@alu.ucam.edu (J.Á.C.); mpzafrilla@ucam.edu (M.P.Z.)

**Keywords:** cognitive function, polyphenols, flavonoids, CREB protein, BDNF, memory, fruits and vegetables, cerebral blood flow

## Abstract

Scientific evidence has shown the relationship between consumption of fruits and vegetables and their polyphenols with the prevention or treatment of diseases. The aim of this review was to find out whether the same relationship exists between fruits and vegetables and cognitive function, especially memory, in a young population. The mechanisms by which polyphenols of fruits and vegetables can exert cognitive benefits were also evaluated. These compounds act to improve neuronal plasticity through the protein CREB (Camp Response Element Binding) in the hippocampus, modulating pathways of signaling and transcription factors (ERK/Akt). In the same way, brain-derived neurotrophic factor (BDNF) is implicated in the maintenance, survival, growth, and differentiation of neurons. All these effects are produced by an increase of cerebral blood flow and an increase of the blood’s nitric oxide levels and oxygenation.

## 1. Impact of the Consumption of Fruit and Vegetables on Health

A healthy lifestyle and nutritional habits are essential for the maintenance of a healthy state in a young population. These habits should include adequate intake of fruits and vegetables [1,2], habitual consumption of omega series fatty acids (about 250–500 mg/day of 6-3/Ω) [3], and limiting the consumption of saturated fatty acids over 10%. Apart from physical activity, adequate intake of vitamins, minerals, and other bioactive compounds through the consumption of fruits and vegetables is essential. In fact, consumption of these foods is related to better aging conditions and a lowered risk of chronic diseases, cancer, physical dysfunction, and mental illness [4,5]. One of the dietary patterns most in line with these guidelines is the Mediterranean diet, characterized by its high content of antioxidants and polyphenols [6,7,8,9]. Recent publications have revealed the association of grain, fruit, and vegetable intake with a decreased risk of cognitive impairment [10].

Consumption of fruits and vegetables in adequate amounts has a direct protective impact on health and is often included in the treatment plans of certain diseases such as obesity [11]; chronic degenerative diseases [12,13]; cardiovascular diseases, through the regulation of cholesterol [14]; the decrease of lipid cell damage in leukocytes and erythrocytes [15]; and functional recovery after stroke and enhanced cognitive function in a parallel block-randomized clinical trial [16]. These impacts have been demonstrated in a recent umbrella review of observational studies [17] and in studies with mice for cerebral ischemia-reperfusion injuries [18].

Several recent meta-analyses have provided evidence supporting a positive relationship between the consumption of fruits and vegetables and prevention of type 2 diabetes mellitus [19], cancer risk [20], and mortality in general [21].

However, other authors have found evidence of this relationship between fruit and vegetable intake and health with only some diseases, such as colon and rectal cancer, hip fracture, stroke, depression, and pancreatic diseases, and limited associations with other outcomes [17]. Bioactive compounds present in fruits and vegetables have a great capacity to neutralize free radicals, averting the deterioration of aging and degenerative diseases. The main proven effects are decreasing oxidative injury [22], and exerting anti-inflammatory and neuroprotective effects [23,24].

Bioactive compounds vary for different fruits and vegetables. For example, pomegranate shows a higher antioxidant capacity than apple [25], presenting similar values to different types of berries, especially members of the Rosaceae and Ericaceae families. The bioactive compounds in berries contain mainly phenolic acids, flavonoids, such as anthocyanins and flavonols, and tannins [26,27]. The effect of bioactive compounds also depends on the size of the portion consumed [28] and the frequency of consumption, as reported in a 12-week study in humans. In this case, the result was significant for memory and cognitive benefits in adults [29].

The mechanisms of action of bioactive compounds have been reported in different studies. Some authors consider that metabolites pass to the colon, where they are metabolized by the action of the local microbiota, leading to small phenolic acids and aromatic catabolites that are absorbed into the circulatory system [30,31,32,33]. In all cases, the determined health benefits must be proved by well-controlled, in vivo intervention studies [34].

The young population has significant neuronal plasticity, showing a high rate of cellular replacement. For this reason, their dietary patterns include foods which have high contents of flavonoids, in order to improve such capacity. Moreover, age cognitive impairment is averted in these cases [35,36,37].

In the elderly population, the susceptibility to oxidative damage is especially increased in the brain. Similarly to young adults, the consumption of flavonoids averts the damage caused by aging. One of the mechanisms responsible might be the flavonoids’ ability to increase cellular signaling and neuronal communication. A high daily intake of fruits and vegetables leads to higher antioxidant levels, lower levels of biomarkers of oxidative stress, and better cognitive performance than healthy subjects consuming low amounts of fruits and vegetables [13,38].

Thus, flavonoids are considered important in cognitive function, and although the mechanisms of action have not yet been clarified, it is known that these compounds modulate cerebral blood flow, inducing changes in processing of memory.

## 2. Polyphenols

Polyphenols are widely distributed in fruits and vegetables and other dietary sources such as nuts, whole grains, olive oil, and infused beverages such as tea. The most common classification of polyphenols is according to the presence or absence of a flavonoid skeleton, classifying them into flavonoids and non-flavonoids [31,39,40].

Flavonoids include anthocyanins [41], which are major polyphenols in berries and red fruits such as grapes [23], catechins [42], proanthocyanidins, flavonols, highly present in green tea and cocoa [43,44], and stilbenes such as resveratrol [45,46]. Other flavonoids are quercetin and kaempferol, which are present in onions, leeks or broccoli. Flavones can be found in high concentrations in parsley or celery, while isoflavones are major polyphenols of soy. Moreover, hesperidin is a major compound in citrus fruits, and cereals such as sorghum [47].

### Protective Capacity of Polyphenols

Polyphenols are able to reduce oxidative stress and cell damage [48,49]. Consistent with this finding, a study where rats were fed with blueberries [50] showed a decrease of oxidative DNA damage, along with lipid peroxidation which was measured by F2-isoprostanes and an increase of reduced glutathione. On the other hand, in 2012, another study reported a similar effect with vegetable soup with a high content of carotenoids. In that case, the results were not the same regarding oxidative damage, except the levels of glutathione, which were also reduced after four weeks of consumption [51].

In chronic inflammatory processes, there is a loss of barrier effect and an overproduction of oxidants such as eicosanoids, cytokines, chemokines, and metalloproteins. The neuroinflammatory process is carried by activated resident glial cells (astrocytes and microglia) and determined by accumulation of circulating immune cells and releasing of proinflammatory cytokines, including tumor necrosis factor (TNF)-α, IL-6, nitric oxide, and reactive oxygen species (ROS) [52]. Polyphenols work to inhibit the release of cytokines, inhibit NADPH oxidase activation and consequent ROS generation in activated glia, and modulate the response to predisposition to chronic inflammation, such as in rheumatoid arthritis and psoriatic diseases. The levels of these mediators contribute to clinical symptoms [53] and modulate cellular signaling processes such as cellular growth and differentiation mediated IL-8 and TNFα through the activation of NF-kB and AP-1 transcription factors.

In neurodegenerative diseases, the presence of those inflammatory biomarkers is related to cognitive impairment [54,55,56] and osteoarthritis by mediation of nitric oxide synthase, cyclo-oxygenase, and metalloproteinases [57]. That prevention of neuroinflammation related to polyphenols has been confirmed with isoflavones recently, and these can cross the blood–brain barrier, making them desirable agents for the prevention of neurological symptoms by inhibiting proinflammatory cytokines, such as TNF-α and in IL-6, mitigating microglial activation, and preventing neuroinflammation. [58]. This inflammatory response has also been studied in muscle damage and homeostasis in intense physical resistance exercise. In a recent study, it was demonstrated to have an effect on post-race muscle damage and inflammation attenuation before and after a marathon race [59]. Moreover, this effect was also reported in young adults after the consumption of vitamin C present in fruits and vegetables through measurement of plasmatic inflammatory markers [60].

Finally, a cross-sectional study revealed the benefits related to the intake of fruit and vegetables on biomarkers of inflammation C-reactive protein, TNF-α, and in IL-6 in adolescents, decreasing F_2_ isoprostane urinary levels [61]. Urinary neuroprostanes related to oxidative attack to the central nervous system (CNS) were reduced in elite athletes by the intake of lemon/aronia juice over 45 days, compared with a placebo rich in polyphenols, through the determination of F_2_ isoprostanes and F_4_ neuroprostanes like markers of oxidative stress in the CNS. Polyphenols reduce oxidative injury in neuronal membrane [62]. ROS are particularly dangerous in the CNS and are related to age and neurodegenerative diseases. In fact, another study revealed that supplementation with polyphenols decreased the effects of oxidative attack in the CNS and improved neural communicability through the consumption of blueberries and Concord grape [42].

Moreover, polyphenols reduced oxidative attack with moderate consumption of red wine, due to their ability to pass the blood–brain barrier, as demonstrated in an intervention measuring docosahexaenoic acid (DHA) peroxidation and production of F_2_-dihomo-IsoPs and neuroprostanes as CNS oxidative injury markers of damage in the central nervous system, in that case hydroxitirosol [63]. However, due to the very low concentration of flavonoids detectable in the brain, it is unlikely that direct antioxidant action can entirely account for cognitive effects in vivo, according to other authors [64]. In fact, neurobiological effects are now believed to be mediated by actions involving the ability to protect vulnerable neurons, enhance neuronal function, and stimulate regeneration via interaction with neuronal intracellular signaling pathways involved in neuronal survival and differentiation, long-term potentiation, and memory [65].

## 3. Cognition

### 3.1. Cognitive Functions

Various cognitive functions, recognized by validated tests of the European Agency for Food Safety (EFSA) [66,67] are as follows:

- Sustained attention: the ability to maintain attention on a stimulus or a task for a long period.
- Selective attention: the ability to focus the mind on a task or a specific stimulus.
- Immediate memory: the ability to maintain a small amount of information in a short time period.
- Working memory: the set of processes that allow the storage and management of information for the performance of complex cognitive tasks such as language, reading, and mathematics.

### 3.2. Cognitive Impairment

Cognitive impairment is normally associated with advanced age or with degenerative diseases affecting cognition such as Alzheimer’s disease or dementia [44,48,56,68,69,70]. The World Health Organization (WHO) estimates a new case of dementia every 4 seconds, which reveals the urgency of the global health crisis associated with cognitive impairment.

The global cost in 2010 in United States was estimated to be $604 billion. This figure equated to around 1% of the aggregated world gross domestic product (GDP) [71]. It is estimated that 10.5 million people in Europe have dementia, accounting for over 22% of the total number of people with dementia worldwide. The total societal costs of dementia in Europe in 2015 were estimated at $301 billion [72]. These diseases are growing in young people, and it has been proven that the conditions can be improved with the consumption of polyphenols through the diet. In fact, as discussed above, polyphenol consumption has been reported in 23 developed countries to decrease the rates of dementia [73], depression [74], and Alzheimer’s disease [75].

For this reason, polyphenols can be considered potential compounds for the treatment and prevention of cognitive impairment [76,77], leading to improvement of the number and quality of synaptic connections in key brain regions [78], especially in the prevention of these pathologies due to the lack of effective symptomatic treatments [79].

In the case of the young population, lifestyle and nutritional habits become extremely important. This population has high basal metabolism requirements. Cell renovation and neuronal plasticity are higher in young people, so their nutritional requirements are more demanding, especially for preventing the loss of cognitive function. The adherence to the Mediterranean diet as a healthy dietary pattern results in the diminution of dementia risk in these young people when they reach an advanced age [80], and socio-economic factors do not affect the adherence to the Mediterranean diet, as shown in this study in Italy [81]. Polyphenol-rich products are beneficial for healthy brain function, particularly in early age, providing acute cognitive benefits. Benefits related to flavonoids and executive function in children have been showed with a recent publication describing positive results in a modified attention network task (MANT) [82,83], although the same author obtained contradictory results three years earlier in a similar research study [84].

On the other hand, related to executive function, other authors have associated the consumption of flavonoids in the young population with decreased risk of developing depression, linking flavonoid interventions with an improved positive mood [85]. This same relationship has also been evidenced by other authors [86,87], especially when it comes to the intake of phenolic acid, flavanones, and anthocyanins. If we talk about individual compounds, we have to especially consider quercetin and naringenin. Recently, a positive effect on the decrease of oxidative stress markers in a dietary pattern with high intake of fruits and vegetables and reduced meat consumption has also been reported [7,88,89].

Adult neurogenesis is the process associated with neural plasticity and the maintenance of normal function in the CNS, and therefore cognitive function and repair of damaged brain cells by aging. Diets enriched with polyphenols have been shown to induce neurogenesis in adult brains in the hippocampal region [90]. Neurogenesis is the process by which brain cells differentiate and proliferate into new neurons, and is a crucial factor in preserving cognitive function and repairing damaged brain cells affected by aging and brain disorders. There is a lack of scientific studies relating to dietary patterns and cognition [35,56] or acute cognitive benefits in the young population [91]. However, different unhealthy diets and lifestyle patterns have been associated with stress in adolescents [92]. In this sense, in Western societies, the trend to prevent or reverse childhood obesity problems by introducing morning and afternoon snacks may also ensure that young people have sufficient energy to complete daily cognitive tasks [93]. It is known that cognitive function declines with age [94], and that this is related to the consumption of antioxidants [56,95].

The improvement of cognition through the consumption of fruit and vegetables in subjects in whom this cognition has decreased due to age shows a greater relationship in the case of flavonoids, especially in anthocyanins and flavones [96]. Additionally, diabetic patients suffer greater oxidative stress and reduced antioxidant activity in the brain, and therefore show a greater cognitive impairment. This hypothesis was supported in a recent study with mice fed with grape and blueberry extracts, reporting possible mechanisms of action which may be associated with modulation of brain plasticity [97,98].

Polyphenols are safe treatment molecules for neurodegenerative diseases, presenting an alternative to drugs without any side effects of their therapeutic consumption. Numerous researchers have classified the beneficial effects of healthy food patterns, concluding that fruits and vegetables have immense potential as therapeutic food components [99].

The improvement of cognitive function has been related to nutrition and lifestyle [97], showing that neuronal damage caused by aging can be effectively countered with the supplementation of antioxidants such as polyphenols [38,100]. This fact has been recently reported in two interventional studies related to blueberry, and grape extracts [98,101]. Moreover, a recent pilot study showed a direct relationship between the consumption of blackcurrant polyphenols and the improvement of cognitive processes associated with attention [102].

## 4. Processes and Measuring Substances in Cognition Functions

### 4.1. Proteins CREB and BDNF (Brain-Derived Neurotrophic Factor)

The hippocampus is the major regulator of memory and learning processes (especially in long-term memory). Polyphenols are involved in neuronal plasticity through the protein CREB (Camp Response Element Binding), the protein kinase, and the activation of receptors in synapses, [24,103,104] modulating pathways of signaling and transcription factors. The phosphorylation of these kinases results in the modulation of synaptic efficacy [105,106]. This has been shown in tissue samples of the hippocampus in mice and the analysis of protein CREB [107]. The NF-kB factor is not only involved in inflammation processes, but in learning and the synaptic activity relationship with protein CREB, which is related to memory [108]. Others authors have reported the mechanism of action in the hippocampus and the receptors involved in the regulation. In neurological diseases and oxidative stress, receptors NMDA and GABA are excessively activated compared to normal situations, increasing synaptic plasticity in memory and teaching [109]. GABA is a neurotransmitter directly related to neurological disorders. For this reason, polyphenols have been acknowledged as potential chemical regulators providing an alternative to other pharmacological treatments. They may have a range of effects including relief of anxiety, improvement in cognition, and acting as neuroprotectants and as sedatives [110]. Recently, others authors have reported that treatment with flavonoids minimizes the increase of cytokines after stroke via the GABA receptor [111].

On the other hand, other factor in relation to synaptic plasticity and cognitive function, is Brain-Derived Neurotrophic Factor (BDNF). BDNF is a neurotrophin related to the maintenance, survival, growth, and differentiation of neurons [112]. In fact, BDNF is so essential to the brain’s cognitive processes that deletion of the BDNF gene was found to weaken memory retention and inhibit long-term potentiation [113]. Recent findings have shown neuroprotective effects of quercetin due to the increment of BDNF mRNA [114,115]. Moreover, there seems to be conflicting results about whether this neurotrophin is genetically influenced by neurodegenerative diseases [116]. Indeed, to date there has been no study to clarify whether the reduction of BDNF levels in neurodegenerative diseases is a downstream consequence of the disease process or part of a specific disease mechanism. In a recent study with rats, it was proven that the level of BDNF was reduced in hippocampus by the administration of carob polyphenols in induced chronic stress [117], learning, and memory [118].

### 4.2. Cerebral Blood Flow

Flavonoids, especially anthocyanins, have a positive effect on the brain cells associated with memory and neuronal function, mainly due to the increase of cerebral blood flow [119,120,121]. This increase in the blood flow to the brain causes an improvement in synaptic plasticity, leading to the improvement of cognitive capacities, shown especially in animal studies and with flavonoid-enriched diets containing grape, pomegranate, strawberry, blueberry, cocoa and pure flavonoids, such as quercetin [122,123,124,125,126]. Authors have reported on not only the increase of polyphenol intake, but also physical activity and intake of alcohol and caffeine. Decreases in cerebral blood flow are induced by commonly consumed amounts of caffeine, while alcohol significantly increases cerebral perfusion in a dose-dependent way [127]. Additionally, lifestyle factors, including dietary composition and physical exercise, can significantly increase CBF, thereby improving cognitive performance.

Included in flavonoids, catechins participate in the signaling cascades of the kinase lipid transcription factors like the CREB protein, as well as changes in the vascular system and the brain [56]. In particular, the connections between neurons through the ERK and AKT signaling pathways are increased, promoting neurotrophies such as BDNF [128].

Moreover, in a recent study on mice regarding oxidative stress and neurodegenerative diseases, improvement of cognitive functions after administration of quercetin over 13 weeks was observed. These results showed an inverse relationship between oxidative stress and neurodegenerative diseases, judging by the reduction of BDNF and CREB and modulation of AKT. Quercetin improved cognitive function as well as increasing BDNF, CREB, and AKT when was administrated over 13 weeks [129]. In the same way, other authors have described a positive protective effect against oxidative-stress-triggered cognitive impairment related to the intake of green tea polyphenols [130]. Finally, epigallocatechin gallate, epicatechin, anthocyanins, and pelargonidins are flavonoids able to cross the blood–brain barrier and therefore exert great effect on cognitive function. This cognitive improvement seems to be regulated by the interaction with ERK and AKT pathways, leading to the modulation of CREB factor and regulation of CREB gene expression [104,131].

Furthermore, the epigallocatechin gallate is able to exert neuroprotection against corticosterone-induced neuron damage by restoring the ERK and PI3K/AKT signaling pathways [132]. The relationship not only between these factors and polyphenols has been demonstrated recently, but also between obesity and insulin resistance [133].

The increase in brain blood flow has also been revealed not only by cognitive test, but also by magnetic resonance in healthy young adults and by using near-infrared spectroscopy (NIRS) in a study with epigallocatechin gallate [29,120,134,135]. The mechanism of action associated with vasodilation is the liberation of nitric oxide through nitric oxide synthase with an improvement in endothelial function [136]. It was consistent with a study on consumption of a cocoa beverage rich in flavonols [137], as well as other authors’ work [138]. A major compound related to these effects is epicatechin, the main flavanol present in cocoa, as evidenced in the study with the Kuna Indians of Panama [139]. That study on cocoa flavonoids supported the idea that flavanol-induced acute changes in brain perfusion could underpin the immediate cognitive-enhancing effects [140]. Accordingly, other authors have reported a direct relationship between the consumption of flavonoids and vasodilation due to nitric oxide synthase activation [137].

Thus, flavonoids are considered important in cognitive function, influencing signaling pathways that are involved in processing of memory. However, the mechanisms of action have not yet been clarified [136]. In this sense, other authors have described similar effects related to resveratrol, assessing the effects on cognitive performance and cerebral brain blood and modulating concentration of hemoglobin and cerebral blood flow, checked with cognitive tasks, NIRS, and determination of hemoglobin and deoxyhemoglobin [141].

Flavonoids act within the cell signaling chain of protein kinases, namely PI3 kinase/AKT. These cascades stimulate gene expression and protein synthesis to maintain long-term potentiation, establish long-term memory [106], modulate transcription factors that participate in signal transduction through protein kinase inhibition, and promote the expression of brain-derived neurotrophic factor (BDNF) that is critical for neurogenesis [142]. In fact, chronic administration of polyphenols has this effect on hippocampal memory by phosphorylation of ERK, CREB, and the expression of BDNF [143].

As some authors have described, the hormone 17β-estradiol has a critical role in the ERK signaling pathway, which had not previously been identified. Therefore, neuron-derived estradiol functions may be a novel neuromodulator in the brain, related to the control of synaptic plasticity and cognitive function [144].

### 4.3. Memory

The protective effects of polyphenols regarding cognition and memory improvement have been highlighted in the case of green tea, mainly associating the epigallocatechin gallate with effects in working memory and attention [145]; through metabolites able to reach the brain parenchyma [146,147]; cocoa polyphenols, as shown during a study of psychological stress in rats [44]; and other polyphenols such as quercetin, which have been shown to be useful in the prevention and treatment of degenerative diseases in humans related to memory [148,149]. This effect appears to be defined by a significant decrease in biomarkers of stress and inflammation, such as dopamine and norepinephrine, cortisol, IL-6, and superoxide dismutase. Quercetin improves learning and memory-impairment-attenuating scopolamine-induced cholinesterase activity and the BDNF level in the prefrontal cortex and hippocampus [150].

There seems to be an improvement of cognitive functions in interventional studies regarding consumption of polyphenol-rich foods, especially flavonoids. These effects appear to be obvious in very young or elderly populations in whom the neural growth of hippocampus or neuronal connectivity can be improved [121]. In particular, the region of the hippocampus which is implicated in memory, especially in older adults with neurodegenerative diseases, is the dentate gyrus [149,151]. This fact has been demonstrated with a resveratrol-based nutraceutical in young adults, subjecting them to demanding cognitive tasks and relating them to brain blood flow through the modulation of nitric oxide [135,152] as well as improving endothelial function [153]. In the same way, flavonoid consumption produced an improvement of the adult proliferation rate in hippocampus in adult hippocampal neurogenesis, directly linked to cognition, proving a potent antioxidant capacity [154].

In a prospective study in French adults, memory, executive function, and language were evaluated. It was not possible to determine a clear and evident association between improvement of these functions and the consumption of fruits or vegetables. However, that relationship did occur with the improvement of cognitive impairment [37]. Likewise, the consumption of multivitamins and their composition has been linked to improvements of cognitive performance in some tasks associated with different types of memory [155].

Despite the above, randomized scientific studies reporting on polyphenols as potential agents on memory and learning seem to be more focused on elderly people (especially related to neurodegenerative diseases) than on young people [104,156] especially in Alzheimer’s disease [157,158].

## 5. Conclusions and Future Perspectives

Large amounts of previous literature have demonstrated the positive effect of polyphenols related to neuroprotection and antioxidant and anti-inflammatory capacity. As regards memory and cognitive functions, other authors have reported interventional studies showing their improvement by increased brain blood flow and BDNF- or CREB-protein-related pathways.

In this context, an adequate intake of fruits and vegetables is necessary for the maintenance of normal cognitive functions. The population with cognitive deterioration has been increasing around the world since the second half of the 20th century. A further decline in cognition might be preventable in the early stages of cognitive impairment by regular intake of polyphenols present in foods. The mechanisms of flavonoids have been shown through the inhibition of free radicals and modulation of signaling pathways that are implicated in cognitive and neuroprotective functions [52,159].

In order to reach an adequate consumption of polyphenols, nutraceuticals are food that can provide an easy and simple way to consume the recommended daily allowance of polyphenols, although more knowledge is necessary about their actual effects on human health bioavailability, pharmacokinetics, and mechanisms of action [160,161]. The nutraceutical industry is becoming the new food industry due to the incorporation of these bioactive substances into our daily lives [162,163].

Additionally, there is a lack of effective pharmaceutical treatments for age-related cognitive decline [164], but an increase of polyphenol-rich nutritional supplements and botanical extracts could be an alternative [76,165].

Related to cognitive impairment, these supplements and vegetarian foods are good options for the improvement of mood and cognitive function in the healthy population. The importance of food supplements and nutraceuticals includes economic factors. A food supplement as cognitive deterioration treatment that would reduce the deterioration by 1% per year could reduce the monetary cost of long-term treatment of patients with cognitive dysfunctions. Therefore, fruits and vegetables could be main foods for the improvement of cognitive functions [96].

Cognitive function has been associated with the consumption of fruit and vegetables in patients with degenerative diseases However, there is a lack of information about interventional studies in humans under clinical conditions [54,166] in healthy, young populations [167], especially related to dietary patterns and cognition [35], reporting neurological benefits associated with individual intake of fruit and vegetables or their synergic effect [4]. There have also not been enough intervention studies in humans about the gut–microbiota–brain axis, which has been proven to be one of the lines by which diet may improve the development and healthy functions of the brain [33].

Therefore, more studies in young and healthy populations seems to be relevant not only for these populations, but also to find out the consequences of chronic consumption of fruits and vegetables on cognitive functions, thus implementing primary prevention actions in young people to minimize or slow down the cognitive impairment caused by age.
